# The dynamic evolutionary history of the bananaquit (*Coereba flaveola*) in the Caribbean revealed by a multigene analysis

**DOI:** 10.1186/1471-2148-8-240

**Published:** 2008-08-22

**Authors:** Eva Bellemain, Eldredge Bermingham, Robert E Ricklefs

**Affiliations:** 1 Smithsonian Tropical Research Institute, Apdo 2072, Balboa, Panama; 2 Department of Biology, University of Missouri-St Louis, 8001 Natural Bridge Road, St Louis, MO 63121-4499, USA

## Abstract

**Background:**

The bananaquit (*Coereba flaveola*) is a small nectivorous and frugivorous emberizine bird (order Passeriformes) that is an abundant resident throughout the Caribbean region. We used multi-gene analyses to investigate the evolutionary history of this species throughout its distribution in the West Indies and in South and Middle America. We sequenced six mitochondrial genes (3744 base pairs) and three nuclear genes (2049 base pairs) for forty-four bananaquits and three outgroup species. We infer the ancestral area of the present-day bananaquit populations, report on the species' phylogenetic, biogeographic and evolutionary history, and propose scenarios for its diversification and range expansion.

**Results:**

Phylogenetic concordance between mitochondrial and nuclear genes at the base of the bananaquit phylogeny supported a West Indian origin for continental populations. Multi-gene analysis showing genetic remnants of successive colonization events in the Lesser Antilles reinforced earlier research demonstrating that bananaquits alternate periods of invasiveness and colonization with biogeographic quiescence. Although nuclear genes provided insufficient information at the tips of the tree to further evaluate relationships of closely allied but strongly supported mitochondrial DNA clades, the discrepancy between mitochondrial and nuclear data in the population of Dominican Republic suggested that the mitochondrial genome was recently acquired by introgression from Jamaica.

**Conclusion:**

This study represents one of the most complete phylogeographic analyses of its kind and reveals three patterns that are not commonly appreciated in birds: (1) island to mainland colonization, (2) multiple expansion phases, and (3) mitochondrial genome replacement. The detail revealed by this analysis will guide evolutionary analyses of populations in archipelagos such as the West Indies, which include islands varying in size, age, and geological history. Our results suggest that multi-gene phylogenies will permit improved comparative analysis of the evolutionary histories of different lineages in the same geographical setting, which provide replicated "natural experiments" for testing evolutionary hypotheses.

## Background

We previously used mitochondrial DNA analyses to describe the history of population expansion and divergence of the bananaquit (*Coereba flaveola*), the most abundant and widely distributed songbird in the West Indies, which also occurs in the humid tropics of continental America [1-3]. The geographic distribution and relationship of mtDNA RFLP haplotypes suggested that populations of this species were periodically invasive, and then quiescent. Here we increase our sample of populations and genes to dissect more finely the population history of bananaquits.

The bananaquit is a small nectivorous and frugivorous emberizine bird (order Passeriformes) that is an abundant resident throughout the West Indies, except for Cuba [4-6]. On the continent, it is widely distributed from southern Mexico through much of South America. Geographic variation in plumage coloration led to the recognition of 41 subspecies throughout the range of the bananaquit [7], but mtDNA RFLP analysis failed to support many of these distinctions [3]. The mtDNA analyses also revealed levels of genetic divergence between some populations within the range of genetic differences observed between species [8,9].

Building on our earlier studies of the bananaquit [1-3], we have now included representatives of more populations from the West Indies and the continent, expanded our nucleotide sample of the mitochondrial genome of each individual to 3744 base pairs, and have added at least 2049 base pairs representing three nuclear gene regions. Our phylogenetic analysis is rooted by the addition of five individuals of three species of emberizid finches representing three genera.

Our principal objectives were to determine the ancestral area of *C. flaveola*, to infer the number and directions of colonization events within the West Indies and between the islands and the continent, and to assess the power of nuclear versus mitochondrial genes to inform analyses of colonization history and subsequent population dynamics. Our phylogenetic results locate the geographic ancestral area of the bananaquit in the region of the Greater Antilles and Bahamas, and strongly support the hypothesis that continental bananaquits were derived from West Indian ancestors. In addition, comparisons of mitochondrial and nuclear gene genealogies exposed a complex and dynamic history of bananaquits in the Greater and Lesser Antilles that would have remained masked without recourse to both maternal and biparental genealogical markers.

## Results

### Sequencing, nucleotide composition and saturation

**Figure 1:**
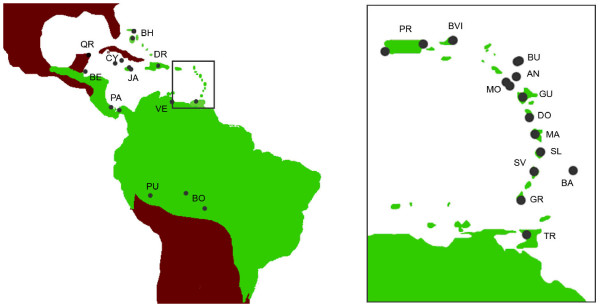
**Geographic representation of the sample localities of the *Coereba flaveola *specimens analyzed**. Locality codes are the same as in additional file 1.

We obtained clean sequences for all 43 *C. flaveola *distributed within the West Indies and South-central America (Figure 1) and for six outgroup samples, with a few exceptions, for six mitochondrial genes and three nuclear genes (see additional file 1). All sequences were submitted to Genbank (accession numbers from EF567429 to EF567954; additional file 1).

Cloning was performed on nine samples for BFib5, 10 samples for Rag1, and two samples for CHDZ. Cloning error rate was estimated to be about 0.1% and cloning errors were eliminated from cloned alleles.

Several properties of the data were examined to ascertain the utility of the genes for phylogenetic analysis (additional file 2). None of the mitochondrial genes exhibited stop codons when translated into amino acids. A test of homogeneity indicated that sequences had similar base frequencies across taxa (p values for all genes were above 0.99; data not shown). As expected, mtDNA sequences showed an anti-guanine bias, except for ND6, which showed an anti-cytosine bias and is the only one of the six genes encoded by the L strand; all other protein-coding mitochondrial genes are encoded by the H strand.

The majority of variable sites in the mitochondrial datasets were third-position substitutions, as expected for protein-coding genes. The mitochondrial sequence dataset included more variation than the nuclear dataset, with a mean percentage of variable sites ranging from 4.8 to 6.2 for mitochondrial genes compared to 0.3 (RAG1, a protein coding region) to 1.8 for nuclear genes.

We calculated the relative substitution rate between mitochondrial and nuclear data by dividing the net average mitochondrial distance and the net average nuclear distance between the Bahamas individuals and the rest of the *C. flaveola *phylogeny. The mean substitution rate was approximately 16 times slower for nuclear genes than for mitochondrial genes.

Plots of the transition/transversion ratio against uncorrected distances indicated that nucleotide substitution in the mitochondrial genome was not saturated (data not shown). All plots were linear whether plotted by position or by gene. Thus, all nucleotides were employed in the ensuing phylogenetic analyses of *C. flaveola*.

### Phylogenetic analyses: outgroups

The closest relatives to *C. flaveola *[10] chosen here as outgroups to root our phylogenies (i.e. *Tiaris olivacea, Loxigilla portoricensis *and *Melanospiza richardsoni*) were divergent by at least 11.2% from a mitochondrial perspective and at least 2% from a nuclear perspective.

### Phylogenetic analyses: congruence among tree topologies using different approaches and different genes

**Figure 2:**
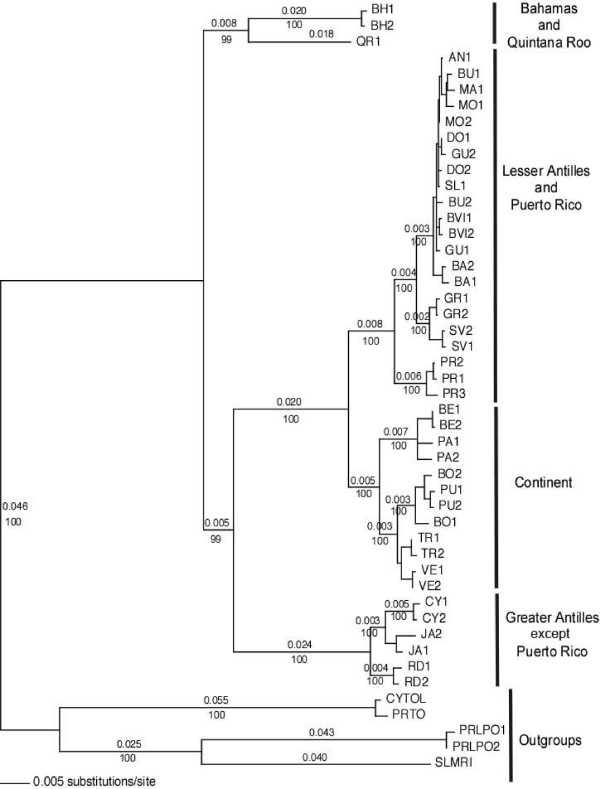
***Coereba flaveola *phylogenetic relationships based on the combined mitochondrial dataset (ATPase 8/6, cyt b, BCO1, ND2 and ND6 genes) inferred from a Bayesian analysis and rooted using three outgroup species (*Tiaris olivacea*, *Loxigilla portoricensis *and *Melanospiza richardsoni*)**. Values above the major branches represent the number of substitutions per site, estimated as the mean value of the branch in all samples of the Markov chain where the branch appears. Values below each major branch represent posterior probability values. Sample names are composed of a locality code (same as in additional file 1) and a sample number.

**Figure 3:**
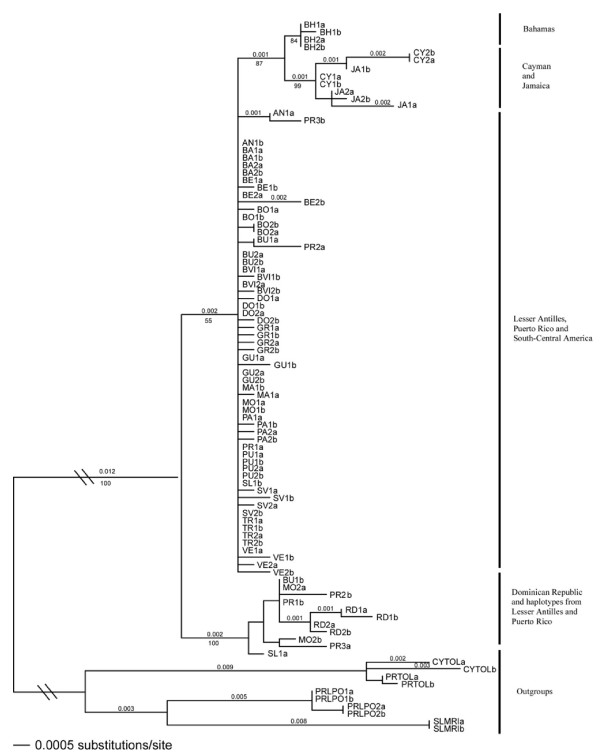
***Coereba flaveola *phylogenetic relationships based on the combined nuclear Bfib5, Rag1 and CHDZ genes, inferred from a Bayesian analysis and rooted using three outgroup species (*Tiaris olivacea*, *Loxigilla portoricensis *and *Melanospiza richardosoni*)**. Letters following each sample name (a or b) represent the two haplotypes for each sample.

Phylogenetic trees generated using the three approaches – MP, ML and Bayesian – were congruent for both mitochondrial and nuclear datasets. We also failed to reject homogeneity of phylogenetic trees based on each mitochondrial gene region independently (p = 0.3) or each nuclear gene region independently (p = 0.07). Therefore, for the subsequent phylogenetic analyses, we combined all six mitochondrial genes for the mitochondrial dataset and all three nuclear genes for the nuclear dataset. Concatenation of the different genes for nuclear data can be problematic as independent sorting of alleles in different parts of the genome and stochasticity of lineage sorting may lead to erroneous species trees [11,12]. However, our results should be unaffected by this issue because the nuclear and mitochondrial trees are congruent, with the exception of the mtDNA of Hispaniolan birds (Figures 2 and 3). The GTR+I+G model of evolution was selected using the AIC criterion in Mr ModelTest for both the mitochondrial and the nuclear datasets.

### Phylogenetic analysis using mtDNA

When describing the phylogeographic results, we use the following area name codes: LA = Lesser Antilles; PR = Puerto Rico; GA = Greater Antilles; SCA = South and Central America; BH/QR = Bahamas/Quintana Roo; DR = Dominican Republic (island of Hispaniola); JA/CY = Jamaica/Cayman Islands). A consensus phylogenetic tree based on a Bayesian analysis combining all six mitochondrial genes identified four major mitochondrial clades representing BH and QR, GA (except PR), LA (including PR), and SCA (Figure 2). Relationships were strongly supported with marginal posterior probability values ≥ 0.99 for all 15 major nodes.

The continental (SCA) clade of bananaquits was sister to the PR and LA clades, which were imbedded within the GA (excluding PR) clades. This topology indicates derivation of the continental clade of bananaquits from within the GA. Within the PR/LA clade, sequences from PR had a sister relationship to the rest of the clade, and bananaquits on Grenada and Saint Vincent form a distinct phylogenetic group sister to populations in the northern LA. Within the SCA clade, samples from Central America (Panama and Belize) were grouped and distinct from samples from South America. Within South America, two groups were distinguished: birds from Trinidad and Venezuela and birds from Peru and Bolivia. A sample from Mexico (Biological Station of Los Tuxlas, Veracruz) sequenced with ND2 (M. Miller, pers. comm.) fell within the Central America group (data not shown) when added to our mitochondrial data.

### Phylogenetic analysis using nuclear DNA

**Figure 4:**
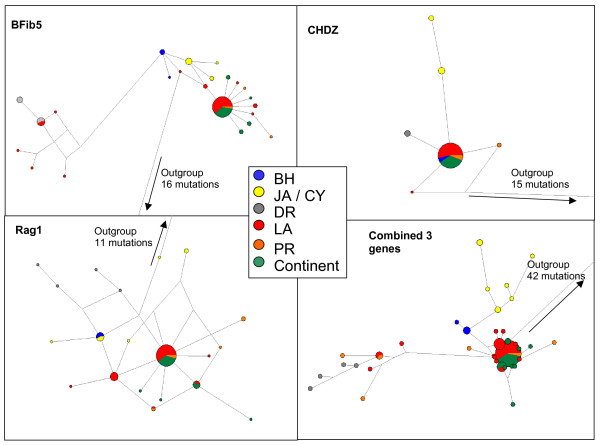
**Haplotype networks for the three nuclear genes (Bfib5, Rag1 and CHDZ) and the combined genes for *Coereba flaveola***. Branch lengths are proportional to the number of mutations and the size of each node is proportional to haplotype frequency. Each of the clades defined from the mitochondrial or nuclear consensus trees (Figures 2 and 3) is given a specific color.

Bayesian analysis combining all three nuclear genes (Figure 3) distinguished four clades: (1) BH, (2) JA and CY, (3) DR together with isolated haplotypes from Montserrat, Barbados, Saint Lucia and PR, and (4) PR, LA and SCA. All three genes showed the affiliation of continental haplotypes with PR/LA haplotypes and the distinctiveness of DR and JA/CY haplotypes. Nevertheless each gene shows a specific pattern. For example, BFib5, the most variable gene, unites the four DR and seven LA/PR haplotypes, a group that is separated from the other sequences by five nucleotide changes. CHDZ, the least variable gene, separates with only one nucleotide change the JA/CY samples on one side and DR samples on the other side from the rest of the phylogeny (Figure 4).

### Mitochondrial versus nuclear phylogenetic analyses

In comparison with the mitochondrial tree, the tree based on nuclear data resolved the more recent divergences poorly. For example, the nuclear data grouped the continental samples with those from the LA. Nonetheless, the nuclear phylogeny was consistent with the mitochondrial tree in the basal branching pattern for *C. flaveola *within the West Indies. The distinctiveness of DR birds in the nuclear tree was surprising given their relative proximity to JA/CY birds in the mitochondrial tree. Because the discrepancy between mitochondrial and nuclear data suggests a distinctive phylogenetic history for the DR population, we sequenced eight additional DR individuals with both mitochondrial (ATPase) and nuclear (BFib5) genes. Those additional sequences confirmed that DR birds grouped with CY and JA birds from a mitochondrial perspective and formed a separate clade from the other populations from a nuclear perspective (data not shown).

### Estimates of population splitting times

The likelihood-ratio test based on the six mitochondrial genes failed to reject the null hypothesis of rate constancy (-ln*L *= 9243.85 enforced tree and 9217.02 non enforced tree, χ^2 ^= 53.66, df = 40, p = 0.073). Therefore, to estimate divergence times, we used linear mtDNA substitution rates of 1.5 and 3% Ma^-1^, which span the range of most calibrations (see "Methods" for justification [13,14]).

The average genetic distance between BH/QR birds and all other populations was 0.0562 ± 0.0037, placing the ingroup root between 1.75 and 3.99 Mya. The average divergence between the GA and LA/PR/SCA groups was 0.0529 ± 0.0038, indicating that the split between those two clades occurred at approximately the same time, i.e., 1.75 to 3.99 Mya. The divergence between the LA/PR clade and the SCA clade was 0.0234 ± 0.0025, implying a split about 0.69 to 1.72 Mya. Finally, PR diverged from the LA approximately 0.34–0.93 Mya.

## Discussion

In this paper, we have described phylogeographic relationships of bananaquit populations to determine the history of colonization within the Caribbean Basin. Additional analyses using coalescent models to infer population parameters require a different sampling regime (i.e., more individuals per population and more nuclear genes) and will be reported separately (Bellemain et al. unpublished).

We provide a well-resolved phylogeny of the bananaquit in the West Indies, based on multiple genes. Aspects of the bananaquit phylogeny highlight points of general interest that are not commonly appreciated for birds. First, we infer that populations sampled in the Greater Antilles and Bahamas were the source of mainland colonization for *C. flaveola*, which is the reverse of the usual continent-to-island direction of colonization. Second, our study demonstrated the dynamic nature of the bananaquit distribution, resulting from several phases of overlapping expansions, especially through the Lesser Antilles. Third, a discrepancy between mitochondrial and nuclear haplotypes among bananaquits on Hispaniola (Dominican Republic samples) suggested the recent replacement of the mitochondrial genome by introgression from Jamaica.

### Mitochondrial versus nuclear data: relative coalescence time, sorting of ancestral polymorphism and inferences concerning phylogenetic history

DNA sequences of mitochondrial genes provide a single estimate of a species phylogeny based on one linkage group [15]. In our analysis of bananaquit phylogeny, we adopted a multi-gene approach based on three nuclear genes as well as DNA sequences for six mitochondrial genes. The more rapid coalescence of mtDNA represents an advantage as it is more likely to resolve nodes with short internode distances, and the probability of incomplete lineage sorting is reduced compared to nuclear DNA [16,17]. However, an evolutionary reconstruction based on multiple independent genes increases confidence that the true species tree has been recovered, and improves phylogenetic resolution by averaging out potentially misleading effects of ancestral polymorphisms [16,18,19]. Furthermore, analytical problems resulting from the stochastic nature of lineage sorting and nucleotide substitution are reduced with an increased sample of genes and nucleotides. Our aim, here, was to explore the evolutionary history of the bananaquit populations within the West Indies from the vantage points offered by the different genes independently and collectively, considering their different mutation and substitution rates, coalescence times, modes of inheritance and effective population sizes.

We obtained a well-resolved phylogeny of *C. flaveola *based on analysis of the six mitochondrial genes. The substitution rate of bananaquit mtDNA was high relative to the observed rate of lineage splitting, as evidenced by abundant synapomorphies and complete lineage sorting (reciprocal monophyly) of the major clades. The data exhibited no saturation and approximated clock-like nucleotide substitution. Thus, we are confident that the tree presented in Figure 2 represents a strongly supported hypothesis of matrilineal relationship.

In contrast, nuclear genes demonstrated considerably lower resolution regarding the phylogenetic relationships of bananaquit populations. The substitution rate of the nuclear genes was, on average, 16 times slower than the mitochondrial genes, and thus closely related clades strongly supported by the mitochondrial data (e.g., north central LA sister to Grenada/St. Vincent, and JA/CY sister to DR) were not distinguished by the nuclear data. Nonetheless, the allelic divergence at nuclear loci and the relative frequency of nuclear alleles across populations (Figure 4) clearly identifies the genetic distinctiveness of the bananaquit clades we have named "Bahamas", "Jamaica" and "Puerto Rico", and lends strong support to the hypothesis that these clades separated early in the diversification of *Coereba*.

The slow nuclear sorting rate relative to population diversification resulted in many examples of incomplete lineage sorting compared to the mitochondrial perspective. Alternatively, the nuclear data could be interpreted as revealing abundant gene flow mediated by male bananaquits, biparental gene flow followed by localized episodes of selective sweeps acting on the mitochondrial genome, or drift associated with reduction in female population size. We suspect incomplete lineage sorting because deep in the bananaquit phylogeny both the nuclear and mitochondrial data consistently identify the same clades, with the single exception that individuals on Hispaniola (DR) have mitochondrial DNA closely related to JA/CY bananaquits, whereas the nuclear markers are allied with those on PR and the north central LA.

### Phylogenetic history of the Dominican Republic population on Hispaniola revealed by the discrepancy between nuclear and mitochondrial data

Nuclear markers separate DR from JA/CY birds, whereas those populations are grouped in the same clade from a mitochondrial perspective. This surprising result was confirmed by sequencing eight additional samples from this population for nuclear (BFib5) and mitochondrial (ATPase) genes (data not shown). Recent introgression of JA/CY mitochondrial haplotypes into the DR population is the most likely explanation of conflicting positions of the DR samples in the nuclear and mitochondrial trees (Figures 2 and 3). Although JA/CY mtDNA completely replaced the local DR mtDNA, we found no evidence of introgression in the nuclear genome. The challenge is to understand the complete introgression of the JA mitotype against a predominately DR nuclear background.

Mitochondrial genomes are particularly susceptible to introgression, although this phenomenon is not clearly understood (see [15] for a review). The ca. four-fold smaller effective population size of the mtDNA genome compared to the nuclear genome implies a higher probability for introgression by mtDNA. However, this outcome would be relatively unlikely if the number of JA immigrants was small relative to the population of resident DR bananaquits. Of course, if immigration from JA coincided with a population crash of Hispaniolan bananaquits, the likelihood of stochastic fixation of the JA mtDNA clade would have increased accordingly.

Alternatively, mitochondrial introgression could have been favoured by selection, while the nuclear genomes of the two populations were incompatible. This type of selection-driven introgression has been demonstrated in *Drosophila *from mtDNA microinjection studies [20] and more recently in wild goats from a molecular phylogenetic study [21]. The authors of the latter study suggested that proto-*Hemitragus *mtDNA could have invaded the ancestral population of *Capra *because the former were better adapted to high altitudes. However, we cannot imagine differences in the selective environments of basal bananaquit populations that might have led to the selective advantage of the JA mitochondrion, considering that the major islands of the Greater Antilles have a similar range of environments.

A third hypothesis is that sexual selection could account for the observation, much in the same manner as hypothesized for *Dendroica occidentalis *warblers along the west coast and islands of British Colombia [22]. In this case, hybrid JA/DR males would have a mating disadvantage, slowing the introgression of nuclear genes relative to the mitochrondrial genomes passed on only by the females. Each backcross generation would dilute the contribution of JA nuclear alleles, while JA mitotypes might become fixed by chance. However, unless the JA mitochondrion experienced a selective advantage, this scenario is likely only in the case of greatly reduced DR populations.

Other biological processes that can create topological incongruence between gene trees include lineage sorting, paralogy, and lateral gene transfer [23]. These alternatives can easily be discounted in the case of DR bananaquits.

First, because several independent nuclear gene sequences support the complete divergence of the nuclear genomes of JA/CY and DR birds, the combination of DR nuclear and JA/CY mitochondrial genomes is not likely the result of incomplete lineage sorting of an ancestral polymorphism in nuclear alleles. Although scenarios implying gene duplications and gene deletions could be inferred for one nuclear gene only, it is highly unlikely that the same scenario may have occurred independently and identically in the three unlinked nuclear genes (RAG-1, Bfib5 and CHDZ). Indeed, the absence of substantial divergence between JA/CY and DR mitochondrial genomes indicates recent derivation. Second, phylogenetic trees using paralogous sequences may be misinterpreted if one assumes that the nuclear sequences are orthologous. Orthologous genes derive from the same locus whereas paralogous genes derive from different loci that originated by gene duplication [24]. However, reconstructing an erroneous gene tree based on paralogy or gene duplication/deletion is highly improbable because it would imply one multiple-gene or genome-wide duplication event at the root of the tree, a panmictic DR/JA/CY population until recently (necessary to explain the similar mitochondrial genomes), and several recent nuclear gene deletions. Finally, lateral gene transfer other than by hybridization/introgression can happen when an organism incorporates genetic material from another distantly related organism, and this can create topological incongruence between gene trees. However, animals seem to be largely unaffected by this phenomenon [25] and it has never been reported for birds.

### The evolutionary history of bananaquits

The mitochondrial phylogeny pictured in Figure 2 confirms the phylogeographic structure of bananaquits revealed by the earlier RFLP analysis [3]. Regional mtDNA haplotype clades displayed levels of sequence divergence typically in excess of 2%, with the largest distances exceeding 5% (e.g., BH versus other populations). The nuclear DNA data confirm the mtDNA-based hypothesis of early divergence amongst bananaquit populations today revealed by three highly differentiated regional assemblages: BH, JA/CY, and PR/LA/SCA.

Bananaquit lineages representing the BH, JA and PR were probably isolated soon after the initial geographic spread of the group in the GA. Subsequent expansions from the BH and JA were geographically restricted. Bananaquits from the BH probably spread to the Yucatán Peninsula of Mexico early in the history of that clade based on the mtDNA sequence of a single bird collected from QR (note that bananaquits from the coast of Veracruz group with other Central American birds, and that the species does not occur elsewhere on the Yucatán Peninsula, except for islands off the coast of Quintana Roo [26]. The sister relationship of this QR individual and the BH sample could place the root of the bananaquit phylogeny on the continent, independently of the widespread and more recent SCA clade. A more likely interpretation is that the QR individual represents relict population of a separate invasion from the Greater Antilles now restricted primarily to Cozumel Island. This raises the intriguing possibility that it is a relict of a former divergent population of bananaquits on Cuba, which would have been a likely source of colonization from the GA to QR. It seems unlikely that bananaquits never existed on Cuba. Their current status has been defined as "possible permanent resident" on cays off the northern coast of Cuba (Ciego de Ávila and Camagüey Provinces) and on the island itself at Gibara (Holguín Province), but breeding has not been established [27]. To summarize, bananaquits from coastal Yucatan deserve additional study, but data in hand suggest that the matriline allied to the BH is narrowly restricted in comparison to the predominant mtDNA haplotype clade in Middle America.

The lineage of bananaquits found today on JA/CY is also narrowly distributed from a joint nuclear and mtDNA perspective. Given the predominance of the JA/CY matriline in the DR, however, it is clear that at least female bananaquits have moved between the islands relatively recently. Thus, migration or range expansion of the JA clade group has been recent in comparison to that of the BH mtDNA haplotype clade.

Nuclear and mitochondrial data indicate that most of the contemporary distribution of bananaquits results from the spread of birds whose descendents are now found in PR, LA and SCA. The geographic expansion of this clade is relatively recent in comparison to the original diversification of bananaquits, and the first split of this expanding lineage (PR clade) separated island from continental birds. Subsequent diversification on the mainland led to reciprocally monophyletic mtDNA haplotype clades, one predominant in South America and the other in Middle America. In the islands, PR populations became isolated from LA populations, and then bananaquits in the north central LA became separated from the populations on Grenada and St. Vincent.

The pattern of evolutionary quiescence followed by geographic expansion is also repeated in the mtDNA record of bananaquits in the LA. This history was clearly revealed in our earlier research [3] based on a mtDNA RFLP analysis of a larger sample of birds per island, and demonstrated that bananaquits had recently expanded their distribution through the northern islands in the chain. However, we did not anticipate the northward expansion of the north-central Lesser Antilles bananaquit clade all the way to the British Virgin Islands. It is particularly noteworthy that bananaquits crossed the Anegada Gap, which serves as a significant biogeographic divide in the West Indies, to colonize islands that would have been connected to PR, or nearly so, during Pleistocene low sea level stands. This seems improbable if the British Virgin Islands had been occupied by the Puerto Rican population of bananaquits, and suggests that a catastrophic event might have caused the extirpation of populations in the northern LA and the British Virgin Islands. The alternative scenario of north-central LA bananaquits displacing PR like birds in the British Virgin Islands as a result of competitive superiority is lessened by the evident failure of this lineage to invade Puerto Rico. Our sampling in PR included coastal areas on the eastern end of the island close to the Virgin Islands.

At the other extreme of the LA, Grenada and Saint Vincent have maintained their evolutionary separation from islands to the north, but no significant separation from one another as measured genetically or morphologically. Both islands harbor melanistic forms of the bananaquit, as well as shared mtDNA haplotypes, that are not found on islands to the north. The evolutionary connection between Grenada and St. Vincent might reflect their separation by a shallow bank of islands (the Grenadines) that would have connected the islands during Pleistocene low sea level stands [28]. This is not the case for other islands in the LA, which are mostly (excluding St. Kitts-Nevis and Antigua-Barbuda) separated one from the other by deep-water channels.

### Phylogenetic support for taxonomic distinctions among subspecies

Our molecular systematic appraisal of bananaquits permits us to assess phylogenetic support for taxonomic distinctions among the many named subspecies. Two subspecies groups were recognized by Paynter [7]: *bahamensis *(from the Bahamas) and *flaveola *(representing all other populations), but most populations in the West Indies have received specific or subspecific epithets at some time in the past, Bond [29] recognizing 16 subspecies. Our data confirm the genetic distinctiveness of the *bahamensis *birds; however, the *flaveola *group encompasses mtDNA haplotype clades that are nearly as divergent as *flaveola *and *bahamensis *groups. For example the JA/CY/DR clade is approximately 5% diverged in mitochondrial sequence from the remainder of the populations recognized as the *flaveola *subspecies by Paynter [7]. Additionally, subspecies such as *portoricensis *on Puerto Rico represent distinct evolutionary lineages. However, more often than not closely related or identical mtDNA haplotypes encompass several subspecies. For instance, the northern LA mtDNA haplotype clade includes four subspecies (*sancti-thomae*, *bartholemica, martinicana*, and *dominicana*), the two Panama bananaquits comprise different subspecies (*columbiana *and *aterrina*), and birds from Bolivia and Peru belong to three different subspecies (*intermedia*, *dispar *and *alleni*). Although finer genetic distinctions consistent with morphological differences between populations may exist, this study adds evidence that presently recognized bird subspecies often do not represent historically and phylogenetically equivalent evolutionary lineages (e.g., [30]).

### Revision of earlier phylogenetic and biogeographic history of the bananaquit

Our analysis also allows us to review the phylogenetic and biogeographic history of the lineage. Bond [31] interpreted the distribution of *C. flaveola *in the West Indies as resulting from two invasions, one from South America spreading north through the LA and west to JA, and the other from Central America spreading north and east to the BH. Our initial mitochondrial RFLP-based survey of bananaquits [3] indicated a different, more complex history. However lacking samples from bananaquit populations on Dominican Republic and the Bahamas, as well as a suitable outgroup, we could not satisfactorily address Bond's [31] hypothesis of a continental origin for the species [3]. Results reported here confirm and extend the earlier molecular phylogenetic analyses.

The sister-group relationship between SCA bananaquit populations rejects Bond's hypothesis, which predicted that the two continental regions should be most closely related to the island populations founded from South and Central America, respectively.

### Islands as the ancestral area of the bananaquit and "reverse colonisation" of the continent

Because species diversity on islands decreases with distance from continental source areas [32,33], biologists have assumed that colonization of islands or archipelagos is a one-way process, and that "reverse colonization" to the mainland against a diversity gradient rarely, if ever, occurs. Our study confirms previous indications that island species also can colonize continental areas. Although the depauperate biotas of remote islands and archipelagos have little chance of recolonizing continental landmasses, near islands, such as the West Indies, have larger biotas with fewer impediments to dispersal, considerably improving the probability of back migration, or onward migration. Colonization from the islands to the mainland has similarly been reported within the West Indies for several bird species (e.g. *Icterus *orioles, [34]; *Myiarchus *tyrant-flycatchers [35]; possibly *Amazona *parrots, [36]). Island to mainland colonization has also been demonstrated in monarch flycatchers from the Solomon Islands to New Guinea/Australia [37] (see [38] for a review).

In the case of the bananaquit, we have provided both mitochondrial and nuclear evidence that permits strong inference that birds from the islands have colonized the mainland. The nesting of the South American to Panama samples within the West Indian clades and the deep paraphyly of bananaquits from the BH and JA clade with respect to all other bananaquits, including all mainland birds (except the single QR individual), represent the strongest lines of evidence that the ancestral node of the extant lineages is most parsimoniously placed in the Greater Antilles. Furthermore, ancestral area analysis (data not shown) based on the Bremer method [39] clearly excludes the continent as the ancestral area of *Coereba*. Finally, near outgroups for the bananaquit are principally emberizid finches from the West Indies [10]. We cannot place the origin (stem lineage) of the species itself, but this issue is not relevant to the present study.

To conclude, islands, including the West Indies, might be significant sources of biodiversity for continents, and we emphasize the importance of considering reverse colonisation for interpreting biogeographic patterns.

### Diversification and range expansion of Coereba flaveola in the West Indies: alternative scenarios

Alternative scenarios of diversification and range expansion, from the Greater Antilles/Bahamas, can explain the phylogenetic patterns observed in both the mitochondrial and nuclear gene trees. They differ mainly in whether the continent was invaded directly from the Greater Antilles or through the Lesser Antilles.

In the simplest scenario, bananaquits expanded from the GA to independently colonize the LA (through PR) and the continent (directly from the GA) approximately 1.7 to 4 Mya, through an Eastern route. Considering the affinity of South American genotypes to those on PR rather than DR or JA, a Western route can be excluded. This scenario is consistent from a mitochondrial point of view. It does not exclude the possibility that several phases of expansion might have occurred within the LA (including a recent one that would have homogenized the gene pool across the northern Antilles). However, direct colonization of South America from GA seems unlikely for several reasons. First, bananaquits, which do not flock and are not strong fliers, would have difficulty making a long over-water flight. Second, virtually all colonization events in the West Indies occur in stepping-stone fashion from one island to the next, rather than over long distances [2,40]. Third, remnants of the nuclear genome of the South American birds in PR and LA (Figure 3) favour dispersal through the islands rather than directly from the GA.

**Figure 5:**
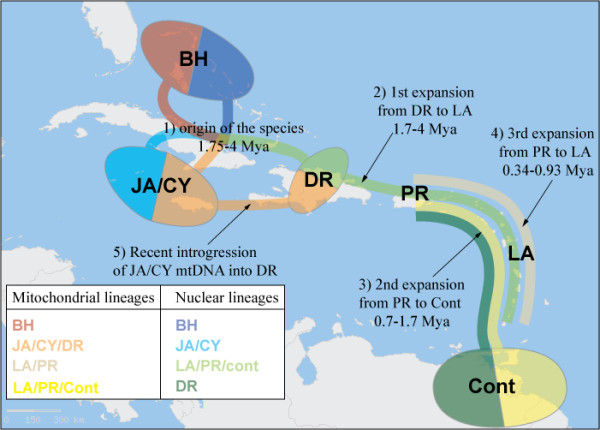
**Phylogeny of *Coereba flaveola *surimposed on the geography of the Caribbean region**. The geographic patterns of the different mitochondrial and nuclear lineages allow resolving the complex phylogenetic history of the species.

We envision colonization from GA to SCA through LA as occurring in the following way (Figure 5). First, bananaquits spread from the DR to PR and through the LA approximately 1.7 to 4 Mya. Evidence of this expansion is seen in the clade of haplotypes in the nuclear phylogeny recovered from DR, PR, and LA. This expansion did not reach the continent of South America.

A second expansion would have occurred from PR through the LA to SCA about 0.7 to 1.7 Mya, as seen in the phylogenetic link between SCA, PR, and LA populations, exclusive of other GA populations, particularly DR (Figure 2). This is also corroborated by the fact that the combined nuclear genes (Figure 4) show the affiliation of continental haplotypes with LA and PR haplotypes, to the exclusion of DR haplotypes. It is also possible that the DR/PR/LA and PR/LA/SCA clades represent the same expansion, followed by lineage sorting.

A third, more recent expansion from PR through the LA, but not to the continent, is indicated by the shared mitochondrial haplotypes of those two populations about 0.34 to 0.93 Mya. After the first and second expansions, PR birds would have had sufficient time to diverge from DR and continental populations from a mitochondrial perspective, but incompletely from a nuclear perspective. This would explain why the LA clade does not include DR mitochondrial sequences but does retain remnants of the DR nuclear genome.

## Conclusion

Analysis of genetic diversity in the bananaquit shows that the different rates of nuclear and mitochondrial genome processes, and incongruities between nuclear and mitochondrial phylogenies, can be used to dissect the often complex phylogeographic history of populations. We emphasize the importance of including multiple genes with distinct evolutionary histories. Our study is the first, to our knowledge, to demonstrate such biogeographic complexity for a single species. First, we demonstrated that the ancestral node of the extant bananaquit lineages is most parsimoniously placed in the Greater Antilles, indicating that islands, including the West Indies, might be significant sources of biodiversity for continents. We suggest that the "reverse colonisation" phenomenon deserves more systematic attention in phylogenetic analyses, and assessing its significance is of prime importance in studies of biogeography and ecology. Second, several phases of colonization and mixing among islands occurred before the bananaquit populations became isolated long enough to develop reciprocal monophyly in mtDNA. Third, the mixed nuclear and mitochondrial genomes in the Dominican Republic and the remnants of old Dominican Republic nuclear lineages in the Lesser Antilles suggest that expanding bananaquit populations mix with resident populations during expansion phases. Thus, genetic compatibility is maintained over periods exceeding three million years and perhaps much more, despite morphological and song differentiation. This does not exclude the possibility that expansions can follow extinctions of local populations or that colonists can competitively exclude local populations, as suggested by non-overlapping distributions of recently expanded mtDNA lineages in several species of Lesser Antillean birds [41].

## Methods

### Sampling

We obtained tissue or blood samples from one to three *C. flaveola *individuals from 16 islands within the West Indies (Antigua, Bahamas, Barbados, Barbuda, British Virgin Islands, Cayman Islands, Dominica, Dominican Republic, Grenada, Guadeloupe, Jamaica, Martinique, Montserrat, Puerto Rico, Saint Lucia and Saint Vincent) and seven countries in Middle and South America (Belize, Bolivia, Mexico, Panama, Peru, Trinidad and Venezuela) (Figure 1). No voucher specimens were collected in the West Indies because identification of species is not an issue. The sampling locations cover the entire distribution of bananaquits within the West Indies, and are broadly representative of the geographic range of the species in Central and South America. Bananaquits occur only accidentally in Cuba and Florida [42]. To root the bananaquit phylogeny, we used the following outgroups: two individuals of *Tiaris olivacea *(from Puerto Rico, PRTOL and the Cayman Islands, CYTOL), two *Loxigilla portoricensis *(Puerto Rico, PRLPO1, PRLPO2), and one *Melanospiza richardsoni *(Saint Lucia, SLMRI), which are recognized as close relatives of *C. Flaveola *[10]. Altogether, our sampling included 49 individuals. Additional file 1 presents details for sample localities. Blood and tissue collection was non-destructive as described in Seutin et al. [3]. On Grenada and St. Vincent, we sampled bananaquits of the typical yellow form as well as the melanistic form [43-45].

### DNA extraction and sequencing

Total cellular DNA was extracted from most samples using the CTAB extraction procedure described in [3]. The following six mitochondrial genes were amplified: ATP synthase 8, ATP synthase 6 (ATPase 8, ATPase 6, 852 bp); cytochrome b (cyt b, 717 bp), cytochrome oxidase (COI, 651 bp), Nadh dehydrogenase 2 (ND2, 1023 bp), Nadh dehydrogenase 6 (ND6, 501 bp). In addition, we amplified three nuclear genes: the recombination activating gene-1 (RAG-1, 1009 bp [46]), the fifth intron of the beta fibrinogen gene (Bfib5, 545 bp [47]), and the chromo-helicase-DNA-binding protein, which is sex-linked (CHDZ, 495 bp [48]). In total, we sequenced 3744 nucleotides of mitochondrial genes and 2049 nucleotides of nuclear genes. Negative controls were included in all PCR amplifications to confirm the absence of contaminants. Amplification of PCR products was verified by visualization on 1.5% TBE agarose gels stained with ethidium bromide. PCR products were then cut from 1.5% TAE agarose gels and left to incubate for two hours at 46°C in presence of gelase.

Sequencing reactions were conducted with the gel purified products and BigDye chemistry (Applied Biosystems, Foster City, CA, USA), and cleaned using Sephadex G50 purification columns (Sigma). Sequencing reaction products were run on an ABIPrism 3130xl automated sequencer (Applied Biosystems). DNA sequence fragments were edited and aligned using Sequencher software version 4.5.

We cloned samples in which nuclear sequences exhibited more than one heterozygous nucleotide site (double peaks of approximately the same height present on both strands) or double peaks extending through a portion of the entire sequence due to allele length variants. Cloning was accomplished using the Promega cloning kit (pGEM-T Easy Vector System II). Ligated plasmids were used to transform *Escherichia coli *competent cells (JM109) selected for inserts by growth on LB plates with IPTG and X-Gal (5'-3'). Positive colonies were grown overnight in LB and up to eight colonies were isolated in 50 μL of purified water. PCR, cycle sequencing, and purifications were then performed as previously described. For some individuals we sequenced one colony per clone and used the resulting sequence to deduce the sequence of the second allele from the original sequence based on the PCR amplification of genomic DNA. Where the original sequence was not interpretable, we sequenced between three and six colonies per clone to obtain clean sequences for both alleles. Cloning errors were identified by comparing multiple sequences and their consensus, and the error rate was estimated by dividing the number of cloning errors by the number of nucleotides sequenced.

Our preliminary data indicated discordant nuclear and mitochondrial trees for Dominican Republic bananaquits (see results), which warranted increased sampling of individuals and genes. Accordingly, we sequenced the mitochondrial ATPase and nuclear BFib5 genes for eight additional birds from diverse locations across the Dominican Republic (additional file 1)

### Phylogenetic analyses

Nucleotide composition, nucleotide bias, percentage of variable sites and parsimony informative sites for both mitochondrial and nuclear genes were analyzed using PAUP* and Sequencher 6.1 [49]. We assessed saturation in the mitochondrial dataset by plotting the transition/transversion ratio against uncorrected genetic distance for the combined mitochondrial dataset.

Phylogenetic analyses for each mitochondrial gene independently, for each nuclear gene independently, and for combined datasets followed verification of the homogeneity of gene regions in combination. Congruence among tree topologies generated using the six mitochondrial genes combined or among the three nuclear genes combined was tested with the partition homogeneity test in PAUP*, with 1000 heuristic replications [50,51]. In turn, we used three approaches to infer relationships among bananaquits. Maximum parsimony (MP) analysis was performed using heuristic searches with TBR branch swapping and support for interior nodes was assessed using a bootstrap approach (1000 replicates). Maximum likelihood (ML) analyses used MrModelTest v2.2 [52] to select the best-fit nucleotide substitution model for the data. Bayesian phylogenetic analyses were conducted using MrBayes v.3.0b4 [53]. We estimated posterior probability distributions by allowing six incrementally heated Markov chains to proceed for two millions generations, with samples taken every 1000 generations. The Markov chain of interest was considered to have converged when stationarity was reached, which we determined by plotting the posterior probability values of nodes against generation time in a cumulative fashion. Burn-in samples were discarded from the two separate analyses, and the remaining samples were combined to create a consensus phylogeny and to estimate posterior probability values and branch lengths.

After fixing the topology of the consensus Bayesian tree, we implemented a |^2 ^test of log-likelihood ratios to compare the difference in support between trees with and without a molecular clock enforced [54] so that we could evaluate the constancy of nucleotide substitution rate within the combined mitochondrial gene dataset. We analyzed the nucleotide substitution rate only for bananaquit lineages and did not include the outgroups.

We failed to reject the null hypothesis of rate constancy in our data. It was not possible to obtain a robust calibration for the application of the molecular clock, first because passerines are poorly represented in the fossil record, and are difficult to identify to species when they do occur, and secondly because calibration information such as radiometric ages of heterochronous sequences or inferred ages of lineage splitting events [13] are not available for the studied species. However, our aim was to provide an approximation (not precise dating) for times of lineage splitting. Therefore, we used a broad range of linear substitution rates of 1.5–3% My^-1 ^reported for bird studies [13,55]. To estimate divergence times, we considered the net average genetic distance between pairs of clades and its standard deviation, using a Tamura-Nei model calculated using MEGA software, version 3.1 [56] and divided the mean genetic distance by 1.5–3%.

We employed the ancestral area method from Bremer [39] to infer the direction and sequence of island and mainland colonization of *C. flaveola *in the West Indies. This is a cladistic method based only on the topology of the tree, which is used to infer a gain-loss ratio for each branch of a simplified area cladogram. The cladistic analysis was based on the topology of the Bayesian tree for the combined mitochondrial dataset.

We used the software NETWORK 4.111 [57] to estimate gene genealogies for each of the nuclear genes and for the combined set of nuclear genes, and to construct unrooted minimum spanning networks. A partition homogeneity test in PAUP*, with 1000 heuristic replications, was used to test for incongruence among tree topologies generated using the seven nuclear genes.

## Authors' contributions

EvB carried out the molecular genetic studies, participated in the sequence alignment and phylogentic analyses and drafted the manuscript. ElB and RER conceived of the study, and participated in its design and coordination and helped to draft the manuscript. All authors read and approved the final manuscript.

## Supplementary Material

Additional file 1 Table 1. Blood and tissue samples used in this study with sampling locations, geographical coordinates and Genbank accession numbers for each sequenced gene. For nuclear genes, each accession numbers corresponds to an allele sequence. NA stands for "none available" (a clean sequence could not be obtained), NS for "not sequenced", numbers beginning with a " A" refer to previously published sequences. Click here for file

Additional file 2 Table 2. Molecular characterization of the mitochondrial and nuclear genes in *Coereba flaveola*. Click here for file
