# Supplementary material for: The dynamic evolutionary history of the bananaquit (Coereba flaveola) in the Caribbean revealed by a multigene analysis
Source: BMC Evol Biol. 2008 Aug 22;8:240. doi: 10.1186/1471-2148-8-240 (PMC2533019; doi:10.1186/1471-2148-8-240)
Supplement: Additional file 1 — Table 1. Blood and tissue samples used in this study with sampling locations, geographical coordinates and Genbank accession numbers for each sequenced gene. For nuclear genes, each accession numbers corresponds to an allele sequence. NA stands for "none available" (a clean sequence could not be obtained), NS for "not sequenced", numbers beginning with a " A" refer to previously published sequences. [file 1471-2148-8-240-S1.doc]

Additional file 1: Table1

Blood and tissue samples used in this study with sampling locations, geographical coordinates and Genbank accession numbers for each sequenced gene. For nuclear genes, each accession numbers corresponds to an allele sequence. NA stands for “none available” (a clean sequence could not be obtained), NS for “not sequenced”, numbers beginning with a “ A” refer to previously published sequences.

|  |  |  |  |  | **NUCLEAR GENES** | | | **MITOCHONDRIAL GENES** | | | | |
| --- | --- | --- | --- | --- | --- | --- | --- | --- | --- | --- | --- | --- |
| **Sample number** | **Name code** | **Locality** | **Lattitude** | **Longitude** | **Rag1** | **BFib5** | **CHDZ** | **ATPase** | **BCOI** | **CytB** | **ND2** | **ND6** |
| AB-CFA2 | BH1 | Bahamas | -771,166 | 264,558 | EF567429/  EF567430 | EF567613/EF567614 | EF567723/EF567724 | AF382954 | AF383067 | AF382993 | AF383109 | EF567910 |
| AB-CFA3 | BH2 | Bahamas | -774,154 | 250,468 | EF567431/  EF567432 | EF567627/EF567628 | EF567733/EF567734 | EF567529 | EF567571 | EF567822 | EF567869 | EF567916 |
| AN-CFA5 | AN1 | Antigua | -618,746 | 170,479 | EF567433/  EF567434 | EF567619/EF567620 | NA | AY115184 | EF567566 | EF567817 | EF567864 | EF567911 |
| BA-CFA5 | BA1 | Barbados | -595,915 | 131,866 | EF567437/EF567438 | EF567623/EF567624 | EF567725/EF567726 | AF132393 | EF567567 | EF567818 | EF567865 | EF567912 |
| BA-CFA7 | BA2 | Barbados | -595,915 | 131,866 | EF567439/EF567440 | EF567625/EF567626 | EF567727/EF567728 | AF132395 | EF567568 | EF567819 | EF567866 | EF567913 |
| BE-CFA373 | BE1 | Belize | -8,818,334 | 17,483,334 | EF567441/EF567442 | EF567615/EF567616 | EF567729/EF567730 | EF567527 | EF567569 | EF567820 | EF567867 | EF567915 |
| BE-CFA403 | BE2 | Belize | -8,818,334 | 17,483,334 | EF567443/EF567444 | EF567617/EF567618 | EF567731/EF567732 | EF567528 | EF567570 | EF567821 | EF567868 | EF567914 |
| BO-CFA1063 | BO1 | Bolivia | -65,4 | -10,02 | EF567445/EF567446 | EF567629/EF567630 | EF567735/EF567736 | EF567530 | EF567572 | EF567823 | EF567870 | EF567917 |
| BO-CFA12938 | BO2 | Bolivia | -61,23 | -13,57 | EF567447/EF567448 | EF567631/EF567632 | EF567737/EF567738 | EF567531 | EF567573 | EF567824 | EF567871 | EF567918 |
| BU-CFA1 | BU1 | Barbuda | -617,824 | 176,421 | EF567451/EF567452 | EF567633/EF567634 | EF567741/EF567742 | AY115185 | EF567574 | EF567825 | EF567872 | EF567919 |
| BU-CFA15 | BU2 | Barbuda | -618,393 | 176,012 | EF567449/EF567450 | EF567635/EF567636 | EF567739/EF567740 | AY115186 | EF567575 | EF567826 | EF567873 | EF567920 |
| BV-CFA34 | BVI1 | British Virgin islands | -64,5 | 18,5 | EF567453/EF567454 | EF567637/EF567638 | EF567743/EF567744 | EF567532 | EF567576 | EF567827 | EF567874 | EF567921 |
| BV-CFA70 | BVI2 | British Virgin islands | -64,5 | 18,5 | EF567455/EF567456 | EF567639/EF567640 | EF567745/EF567746 | EF567533 | EF567577 | EF567828 | EF567875 | EF567922 |
| CY-CFA-CB319 | CY1 | Cayman islands | -81,25 | 19,333 | EF567457/EF567458 | EF567641/EF567642 | EF567747/EF567748 | EF567534 | EF567578 | EF567829 | EF567876 | EF567924 |
| CY-CFA-GC40 | CY2 | Cayman islands | -798,167 | 198,167 | EF567459/EF567460 | EF567643/EF567644 | EF567749/EF567750 | EF567535 | EF567579 | EF567830 | EF567877 | EF567923 |
| DO-CFA11 | DO1 | Dominica | -61,377 | 15,344 | EF567461/EF567462 | EF567645/EF567646 | EF567751/EF567752 | EF567536 | EF567580 | EF567831 | EF567878 | EF567926 |
| DO-CFA16 | DO2 | Dominica | -61,377 | 15,344 | EF567463/EF567464 | EF567647/EF567648 | EF567753/EF567754 | AY115187 | EF567581 | EF567832 | EF567879 | EF567927 |
| GR-CFA20 | GR1 | Grenada | -617,461 | 120,094 | EF567465/EF567466 | EF567649/EF567650 | EF567755/EF567756 | EF567537 | EF567582 | EF567833 | EF567880 | EF567929 |
| GR-CFA25 | GR2 | Grenada | -617,461 | 120,094 | EF567467/EF567468 | EF567651/EF567652 | EF567757/EF567758 | EF567538 | EF567583 | EF567834 | EF567881 | EF567928 |
| GU-CFA1 | GU1 | Guadeloupe | -616,083 | 161,837 | NA | EF567653/EF567654 | EF567759/EF567760 | AY115191 | EF567584 | EF567835 | EF567882 | EF567930 |
| GU-CFA25 | GU2 | Guadeloupe | -616,083 | 161,837 | EF567469/EF567470 | EF567655/EF567656 | EF567761/EF567762 | EF567539 | EF567585 | EF567836 | EF567883 | EF567931 |
| JA-CFA1 | JA1 | Jamaïca | -779,396 | 180,352 | EF567471/EF567472 | EF567657/EF567658 | EF567763/EF567764 | EF567540 | EF567586 | EF567837 | NA | EF567933 |
| JA-CFA41 | JA2 | Jamaïca | -781,422 | 182,128 | EF567473/EF567474 | EF567659/EF567660 | EF567765/EF567766 | NA | EF567587 | EF567838 | EF567884 | EF567932 |
| MA-CFA2 | MA1 | Martinica | -610,925 | 146,677 | EF567475/EF567476 | EF567661/EF567662 | EF567767/EF567768 | AY115193 | EF567588 | EF567839 | EF567885 | EF567934 |
| MO-CFA1 | MO1 | Montserrat | -622,209 | 167,587 | EF567477/EF567478 | EF567663/EF567664 | EF567769/EF567770 | AY115195 | EF567589 | EF567840 | EF567886 | EF567935 |
| MO-CFA20 | MO2 | Montserrat | -621,942 | 166,854 | EF567479/EF567480 | EF567665/EF567666 | EF567771/EF567772 | AY115196 | EF567590 | EF567841 | EF567887 | EF567936 |
| MX-CFA-QR219 | QR1 | Quintana Roo | -87,422 | 19,422 | NA | NA | NA | EF567547 | EF567596 | EF567849 | EF567893 | EF567945 |
| PA-CFA186 | PA1 | Panama | -80,538 | 87,865 | EF567481/EF567482 | EF567667/EF567668 | EF567773/EF567774 | EF567541 | EF567591 | EF567842 | EF567888 | EF567937 |
| PA-CFA341 | PA2 | Panama | -822,667 | 9,25 | EF567483/EF567484 | EF567669/EF567670 | EF567775/EF567776 | EF567542 | EF567592 | EF567843 | EF567889 | EF567938 |
| PR-CFA11366 | PR1 | Puerto Rico | -671,705 | 180,253 | EF567487/EF567488 | EF567673/EF567674 | EF567777/EF567778 | EF567543 | EF567593 | EF567844 | EF567890 | EF567939 |
| PR-CFA11367 | PR2 | Puerto Rico | -656,536 | 183,368 | EF567489/EF567490 | EF567675/EF567676 | EF567779/EF567780 | AY115198 | EF567594 | EF567845 | EF567891 | EF567941 |
| PR-CFA24 | PR3 | Puerto Rico | -671,705 | 180,253 | EF567485/EF567486 | EF567671/EF567672 | EF567781/EF567782 | EF567544 | EF567595 | EF567846 | EF567892 | EF567940 |
| PU-CFA810 | PU1 | Peru | -73,544 | -10,673 | EF567491/EF567492 | EF567677/EF567678 | EF567783/EF567784 | EF567545 | EF567597 | EF567847 | EF567894 | EF567943 |
| PU-CFA815 | PU2 | Peru | -73,544 | -10,673 | EF567493/EF567494 | EF567679/EF567680 | EF567785/EF567786 | EF567546 | EF567598 | EF567848 | EF567895 | EF567944 |
| RD-CFA1 | RD1 | Dominican Republic | -70,618 | 191,151 | EF567495/EF567496 | EF567681/EF567682 | EF567787/EF567788 | EF567548 | EF567599 | EF567850 | EF567896 | EF567946 |
| RD-CFA2 | RD2 | Dominican Republic | -70,618 | 191,151 | EF567497/EF567498 | EF567683/EF567684 | EF567789/EF567790 | EF567549 | EF567600 | EF567851 | EF567897 | EF567947 |
| RD-CFA20 | RD3 | Dominican Republic | -70,6522 | 191,905 | EF567499/EF567500 | EF567687/EF567688 | EF567791/EF567792 | EF567553 | NS | NS | NS | NS |
| RD-CFA21 | RD4 | Dominican Republic | -69,7954 | 18,3546 | EF567501/EF567502 | EF567689/EF567690 | EF567793/EF567794 | EF567554 | NS | NS | NS | NS |
| RD-CFA22 | RD5 | Dominican Republic | -69,7954 | 18,3546 | NS | EF567691/EF567692 | NS | EF567555 | NS | NS | NS | NS |
| RDCFA24 | RD6 | Dominican Republic | -69,7954 | 18,3546 | NS | EF567693/EF567694 | NS | EF567556 | NS | NS | NS | NS |
| RD-CFA25 | RD7 | Dominican Republic | -69,7954 | 18,3546 | NS | EF567695/EF567696 | NS | EF567551 | NS | NS | NS | NS |
| RD-CFA27 | RD8 | Dominican Republic | -69,7954 | 18,3546 | NS | EF567697/EF567698 | NS | EF567557 | NS | NS | NS | NS |
| RD-CFA28 | RD9 | Dominican Republic | -718,000 | 18,6500 | NS | EF567699/EF567700 | NS | EF567550 | NS | NS | NS | NS |
| RD-CFA6 | RD10 | Dominican Republic | -718,000 | 18,6500 | NS | EF567685/EF567686 | NS | EF567552 | NS | NS | NS | NS |
| SL-CFA16 | SL1 | Saint Lucia | -608,667 | 139,833 | EF567503/EF567504 | EF567701/EF567702 | EF567795/EF567796 | AF132400 | EF567601 | EF567852 | EF567898 | EF567948 |
| SV-CFA2077 | SV1 | Saint Vincent | -612,165 | 131,337 | EF567505/EF567506 | EF567703/EF567704 | EF567797/EF567798 | AF132407 | EF567602 | EF567853 | EF567899 | EF567949 |
| SV-CFA2129 | SV2 | Saint Vincent | -61,235 | 131,954 | EF567507/EF567508 | EF567705/EF567706 | EF567799/EF567800 | AF132411 | EF567603 | EF567854 | EF567900 | EF567950 |
| TR-CFA29 | TR1 | Trinidad | -614,595 | 10,602 | EF567509/EF567510 | EF567707/EF567708 | EF567801/EF567802 | EF567558 | EF567604 | EF567855 | EF567901 | EF567951 |
| TR-CFA32 | TR2 | Trinidad | -614,595 | 10,602 | EF567511/EF567512 | EF567709/EF567710 | EF567803/EF567804 | AY115200 | EF567605 | EF567856 | EF567902 | EF567952 |
| VE-CFA10 | VE1 | Venezuela | -631,167 | 105,633 | EF567513/EF567514 | EF567711/EF567712 | EF567805/EF567806 | EF567559 | EF567606 | EF567857 | EF567903 | EF567954 |
| VE-CFA3 | VE2 | Venezuela | -686,333 | 10,45 | EF567515/EF567516 | EF567713/EF567714 | EF567807/EF567808 | EF567560 | EF567607 | EF567858 | EF567904 | EF567953 |
| CY-TOL-GC79 | CYTOL | Cayman | -81,25 | 19,333 | EF567517/EF567518 | EF567715/EF567716 | NA | EF567561 | EF567608 | EF567859 | EF567905 | EF567925 |
| PR-LPO1 | PRLPO1 | Puerto Rico | 180,828 | -660,639 | EF567519/EF567520 | NA | EF567811/EF567812 | EF567563 | EF567609 | EF567860 | EF567906 | NA |
| PR-LPO26 | PRLPO2 | Puerto Rico | 180,828 | -660,639 | EF567521/EF567522 | EF567717/EF567718 | EF567809/EF567810 | EF567564 | EF567610 | EF567861 | EF567907 | NA |
| PR-TOL2 | PRTOL | Puerto Rico | -656,536 | 183,368 | EF567523/EF567524 | EF567719/EF567720 | EF567813/EF567814 | EF567562 | EF567611 | EF567862 | EF567908 | EF567942 |
| SL-MRI2 | SLMRI | Saint Lucia | NA | NA | EF567525/EF567526 | EF567721/EF567722 | EF567815/EF567816 | EF567565 | EF567612 | EF567863 | EF567909 | NA |
