# Supplementary material for: The dynamic evolutionary history of the bananaquit (Coereba flaveola) in the Caribbean revealed by a multigene analysis
Source: BMC Evol Biol. 2008 Aug 22;8:240. doi: 10.1186/1471-2148-8-240 (PMC2533019; doi:10.1186/1471-2148-8-240)
Supplement: Additional file 2 — Table 2. Molecular characterization of the mitochondrial and nuclear genes in Coereba flaveola. [file 1471-2148-8-240-S2.doc]

## Additional file 2: Table 2

## Molecular characterization of the mitochondrial and nuclear genes in *Coereba flaveola*.

|  | **Mitochondrial genes** | | | | | | **Nuclear genes** | | |
| --- | --- | --- | --- | --- | --- | --- | --- | --- | --- |
|  | **ATPase 8** | **ATPase 6** | **CytB** | **BCO1** | **ND2** | **ND6** | **BFib5** | **CHDZ** | **Rag-1** |
| **Number of nucleotide sites** | 168 | 684 | 717 | 651 | 1023 | 501 | 542 | 492 | 1009 |
| **Number of distinct haplotypes** | 30 | 13 | 24 | 31 | 30 | 31 | 18 | 5 | 19 |
| **Nucleotide composition** |  |  |  |  |  |  |  |  |  |
| % G | 7.01 | 11.61 | 13.46 | 16.82 | 11.08 | 41 | 20.84 | 21.77 | 25.19 |
| % A | 30.2 | 28.18 | 27.4 | 25.64 | 28.29 | 10.5 | 30.23 | 26.55 | 30.7 |
| % T | 24.13 | 23.7 | 22.74 | 24.21 | 23.38 | 37.83 | 31.96 | 35.55 | 23.13 |
| % C | 38.65 | 36.51 | 36.39 | 33.33 | 37.24 | 10.68 | 16.96 | 16.13 | 20.98 |
| **Mean uncorrected “p” distance** | 2.36 | 3.53 | 2.41 | 2.58 | 3.12 | 3.75 | 0.43 | 0.1 | 0.14 |
| **% Variable sites (% informative)** |  |  |  |  |  |  |  |  |  |
| First position | 4.8 (4.2) | 3.4 (3.4) | 1.1 (1.1) | 1.1 (1.1) | 3.3 (3.3) | 4.6 (4.6) |  |  | 0 (0) |
| Second position | 2.4 (1.8) | 0.9 (0.9) | 0.3 (0.3) | 0 (0) | 0.6 (0.6) | 1.8 (1.8) |  |  | 0.7 (0.7) |
| Third position | 10.12 (9.5) | 14.2 (14.2) | 13.1 (13.1) | 16.3 (14.8) | 15.1 (14.2) | 12 (12) |  |  | 0.1 (0.1) |
| Total | 5.8 (5.2) | 6.2 (6.1) | 4.8 (4.8) | 5.3 (5.3) | 6.3 (6) | 6.1 (6.1) | 1.8 (1.8) | 1.1 (1.1) | 0.3 (0.3) |
| **Ti/Tv ratio** | 2.32 | 5.62 | 7.39 | 8.64 | 9.03 | 8.72 | 3.37 | 1.41 | 1.5 |

## Values represent percentages with absolute numbers in parentheses. Ti = transitions; Tv = transversion. The percentage of variable sites per position is mentioned only for protein coding genes.
